# A Retrospective Study of Milligan-Morgan Versus LigaSure Hemorrhoidectomy in the Treatment of Symptomatic Hemorrhoids at an Institute in North India

**DOI:** 10.7759/cureus.66430

**Published:** 2024-08-08

**Authors:** Anant Kaur Virk, Rohin Kansal, Carol Singh, Madhav Mehta, Baninder Arora, Anmol Singh, Kashish Malhotra, Jasneet Grewal, Himel Mondal, Ashvind Bawa

**Affiliations:** 1 Department of General Surgery, Dayanand Medical College and Hospital, Ludhiana, IND; 2 Department of Medicine, Adesh Institute of Medical Sciences and Research, Bathinda, IND; 3 Department of Physiology, All India Institute of Medical Sciences, Deoghar, IND

**Keywords:** return-to-work, piles, general surgery, milligan–morgan, ligasure, hemorrhoidectomy, hemorrhoids

## Abstract

Objective

The present study aimed to assess and compare the surgical outcomes of hemorrhoidectomies performed using two different techniques: conventional Milligan-Morgan and another popular vessel sealing approach of LigaSure hemorrhoidectomy.

Methods

This retrospective study was conducted at our tertiary care hospital and involved patients who underwent either of the surgical procedures from June 2016 to March 2022. The patient demographics and data on the duration of operation, hospital stay, and postoperative recovery were collected and evaluated.

Results

Of the 91 cases reviewed, a total of 44 patients underwent Milligan-Morgan open hemorrhoidectomy and 47 had LigaSure hemorrhoidectomy. The mean operative time was significantly shorter for LigaSure hemorrhoidectomy (33.84 ±9.18 vs. 23.15 ±3.36 minutes for Milligan-Morgan and LigaSure, respectively, p<0.0001). Additionally, in comparison to Milligan-Morgan open hemorrhoidectomy, the LigaSure hemorrhoidectomy group exhibited a significant reduction in hospital stay (2.20 ±0.79 vs. 1.47 ±0.50 days), lower pain score [6.55 ±1.19 vs. 5.30 ±1.10 on the visual analog scale (VAS) on day one and 2.25 ±1.26 vs. 1.47 ±0.78 VAS on day seven], and faster return to normal activities (18.18 ±4.30 vs. 14.85 ±3.15 days).

Conclusions

When pitted against the traditional Milligan-Morgan method, the LigaSure approach to performing a hemorrhoidectomy is superior, owing to the shorter duration of operation, shorter hospital stays, lesser pain, and earlier return to normal activities. In light of these findings, surgeons may consider choosing this procedure to improve surgical outcomes and efficiency.

## Introduction

Hemorrhoidal disease is a common anorectal illness characterized by anal cushion displacement and hypertrophy [1,2]. Chronic constipation, a low-fiber diet, pregnancy, straining during defecation, obesity, and a sedentary lifestyle are all risk factors for this condition, particularly in people over the age of 40 years [3]. Despite a reported incidence of 4.4%, its occurrence is substantially more pronounced yet difficult to assess because of the considerable variability in its presentation [1,4]. The Goligher classification of hemorrhoidal disease is based on the degree of prolapse and reducibility of the hemorrhoid, which helps determine the future course of action [2,5,6].

As for other various pathologies, both conservative and surgical modalities are available for the management of hemorrhoids. The conservative treatment encompasses lifestyle adjustments, dietary changes (increased water and fiber consumption), behavioral therapies, over-the-counter topical medicines, and stool softeners [3,5]. Conservative medicinal treatment is mostly effective for treating grade I and II hemorrhoids, while surgical management is advised for grades III and IV [7,8]. The surgical options include stapled hemorrhoidopexy, Doppler-guided hemorrhoidal artery ligation, harmonic scalpel hemorrhoidectomy, conventional hemorrhoidectomy, and LigaSure hemorrhoidectomy [8,9].

The Ferguson method (closed) or Milligan-Morgan (open) approach are the most commonly employed conventional hemorrhoidectomy techniques [10]. Each of these surgical procedures has its own merits and limitations, limiting the adoption of any single technique as the gold standard. Various clinical studies and meta-analyses have been conducted to compare the efficacy of these surgical approaches [11-14]. Open Milligan-Morgan hemorrhoidectomy is a commonly performed procedure using a diathermy cautery pencil where the symptomatic hemorrhoids are excised. However, it has been frequently associated with postoperative pain, blood loss, and complications with delayed wound healing [15,16]. The LigaSure technique has been hailed as an alternative modality: a faster, safer, and more precise method for bloodless hemorrhoid excision with little tissue damage, minimal infections, and faster wound healing. The LigaSure vessel sealing system is a bipolar electrothermal machine that uses an enhanced combination of pressure and electrical energy to seal blood vessels. It demonstrates coagulation of vessels up to 7 mm with little thermal dissipation, tissue charring, and minimal trauma [13,17].

There is a dearth of studies in the literature in the context of clinical practices in northern India evaluating and comparing the surgical results of these two procedures in terms of operative time, postoperative complications, pain score, hospital stay, and return to routine work and daily activities. Hence, the current study sought to compare the efficacy of LigaSure hemorrhoidectomy vs. open Milligan-Morgan hemorrhoidectomy regarding outcomes in the treatment of grade III or IV hemorrhoids.

## Materials and methods

Study design and setting

We conducted a retrospective analysis of 91 hemorrhoidectomies at the surgical unit of our tertiary care institute by evaluating procedures performed between June 2016 and March 2022. The study was conducted after obtaining approval from the Institutional Ethics Committee. Out of the 91 patients, 44 underwent open hemorrhoidectomy and 47 patients underwent the LigaSure hemorrhoidectomy.

Inclusion and exclusion criteria

Inclusion criteria were adults above 18 years of age suffering from symptomatic prolapsing internal hemorrhoids and medically fit for anesthesia. Patients with hematological disorders, concomitant anorectal abnormalities like anal fistulas and anal Crohn’s disease were excluded from the study.

Procedure

**Figure 1:**
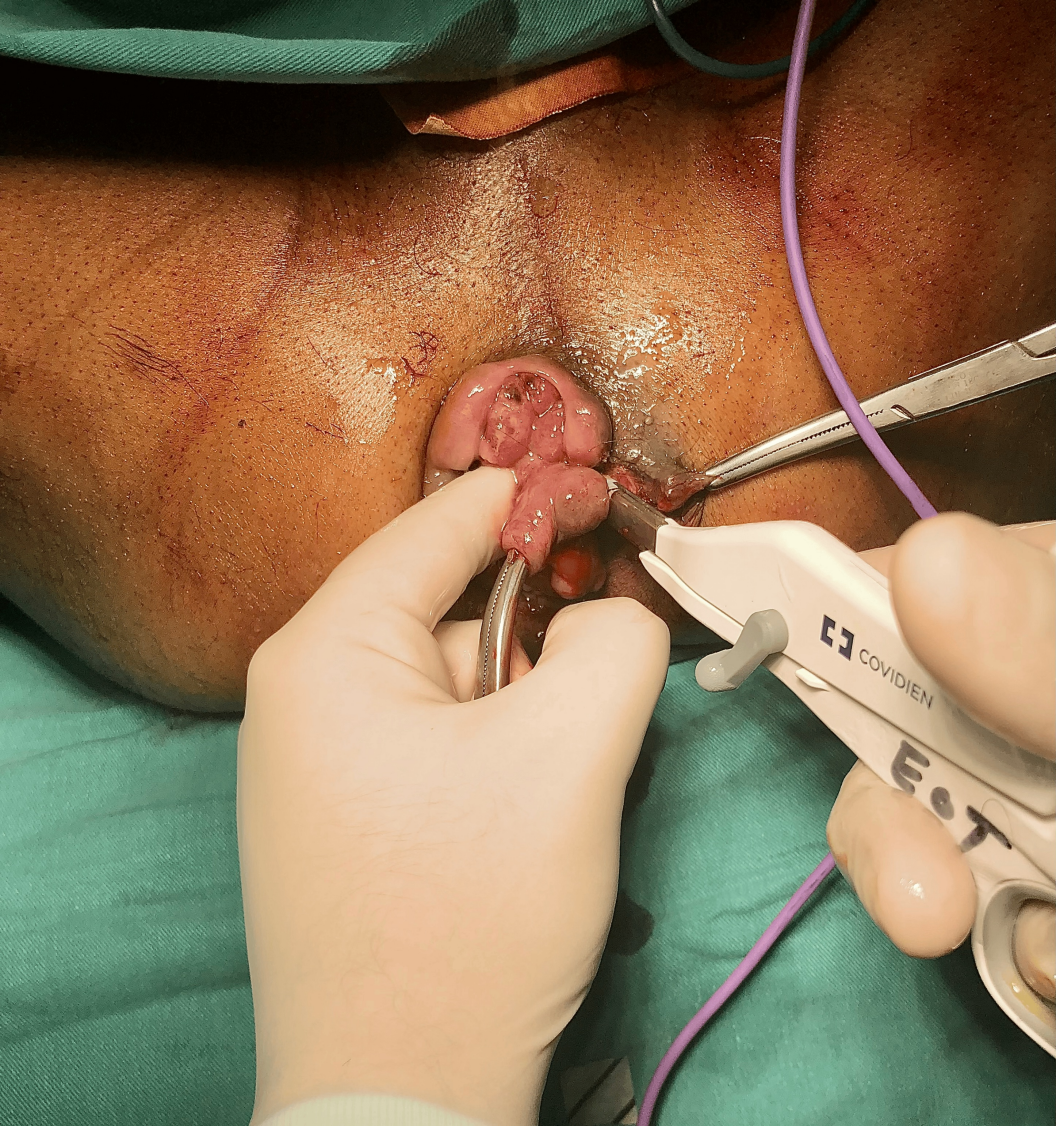
Image showing the use of LigaSure handheld device for hemorrhoidal mass excision

Both groups of patients were admitted on the morning of the operation. Preoperatively, patients in both groups were given a sodium phosphate enema, and all procedures were done under general anesthesia. All surgeries included in the study had been performed by the same surgeon. After creating a V-shaped incision at the mucocutaneous junction, the hemorrhoidal mass was withdrawn with artery forceps in LigaSure hemorrhoidectomy. The hemorrhoidal mass was then excised with the LigaSure handheld device (Figure 1) and the pedicle was ligated with a Vicryl 2-0 suture, and hemostasis was achieved. The wound edges were closed with a 3-0 monocryl continuous suture. A pack was placed toward the end of the surgery and removed the following night. Meanwhile, a diathermy cautery pencil was used to accomplish the excision of hemorrhoidal mass in case of open hemorrhoidectomy. Care was taken to avoid damage to the internal sphincter fibers. The pedicle was ligated using Vicryl 2-0 suture. The edges were left open to heal by secondary intention. A pack was then inserted, which was removed the following night.

Data collection

All patient demographics, grade, number of hemorrhoids, operating time, postoperative problems, pain score [visual analog scale (VAS)], hospital stay, and duration for return to work had been meticulously recorded both during the hospital stay and follow-up visits as part of the routine documentation, with the patients having at least one follow-up visit on day seven from the date of surgery. This data was then made use of in the current retrospective study to draw a contrast between the two procedures under consideration. 

Statistical analysis

SPSS Statistics Version 21 (IBM Corp., Armonk, NY) was used for statistical analysis of the collected data. The numerical results are expressed as mean ±standard deviation (SD). The chi-square test, Mann-Whitney U test, and Student’s t-test were performed for the analysis of significance. A p-value <0.05 was considered statistically significant.

## Results

The study included 91 patients, of which 44 underwent Milligan-Morgan open hemorrhoidectomy (mean age: 49.11 ±15.58 years) and 47 underwent LigaSure hemorrhoidectomy (mean age: 48.83 ±14.77 years). The demographic characteristics are presented in Table 1. There was no significant difference in these characteristics between the groups. However, the gender distribution exhibited a statistically significant difference (p=0.005) between the two groups, with females (male/female: 20/24) dominating the open group while males (male/female: 35/12) constituted the majority in the LigaSure group.

**Table 1:** Comparison of patient characteristics between open hemorrhoidectomy and LigaSure hemorrhoidectomy groups ^*^Statistically significant p-value. ^#^Chi-squared test of significance.^ Z^Mann-Whitney U test of significance. ^t^Student's t-test of significance SD: standard deviation

Characteristics	Open hemorrhoidectomy (n=44)	LigaSure hemorrhoidectomy (n=47)	P-value
Age range, years	26-73	26-83	
Age, years, mean ±SD	49.11 ±15.58	48.83 ±14.77	0.929^t^
Gender, M/F	20/24	35/12	0.005^*#^
Grade III, n (%)	26 (59.1%)	29 (61.7%)	0.799^#^
Grade IV, n (%)	18 (40.9%)	18 (38.3%)	
Preoperative hemoglobin, g/dL, mean ±SD	11.64 ±2.15	11.81 ±1.94	0.885^Z^

The mean operative time was significantly shorter for LigaSure hemorrhoidectomy (33.84 ±9.18 vs. 23.15 ±3.36 minutes in Milligan-Morgan and LigaSure, respectively, p<0.0001). In comparison to Milligan-Morgan open hemorrhoidectomy, the LigaSure hemorrhoidectomy group exhibited a significant reduction in hospital stay (2.20 ±0.79 vs. 1.47 ±0.50 days, p<0.0001), lower pain score (6.55 ±1.19 vs. 5.30 ±1.10 on VAS on day one; 2.25 ±1.26 vs. 1.47 ±0.78 on VAS on day seven, p<0.0001) and faster return to normal activities (18.18 ±4.30 vs. 14.85 ±3.15 days, p<0.0001). Although the number of hemorrhoids ranged from one to four in all patients, the presence of two hemorrhoids was most abundant for both groups, with no statistical significance, as shown in Table 2.

**Table 2:** Assessment of operative and postoperative parameters between the open hemorrhoidectomy and LigaSure hemorrhoidectomy groups ^*^Statistically significant p-value. ^#^Chi-squared test of significance. ^Z^Mann-Whitney U test of significance SD: standard deviation

Parameters	Open hemorrhoidectomy (n=44)	LigaSure hemorrhoidectomy (n=47)	P-value
Number of hemorrhoids, n (%)			
1	5 (11.4%)	7 (14.9%)	0.479^#^
2	17 (38.6%)	22 (46.8%)	
3	15 (34.1%)	15 (31.9%)	
4	7 (15.9%)	3 (6.4%)	
Operative time, minutes, mean ±SD	33.84 ±9.18	23.15 ±3.36	<0.0001*^Z^
Postoperative complications, n (%)			
No complications	39 (88.6%)	45 (95.7%)	0.247^#^
Anal stenosis	0	1 (2.1%)	
Bleeding	4 (9.1%)	1 (2.1%)	
Urinary retention	1 (2.3%)	0	
Days in hospital, mean ±SD	2.20 ±0.79	1.47 ±0.50	<0.0001*^Z^
Return to work, days, mean ±SD	18.18 ±4.30	14.85 ±3.15	<0.0001*^Z^
VAS score, mean ±SD			
Day 1	6.55 ±1.19	5.30 ±1.10	<0.0001*^Z^
Day 7	2.25 ±1.26	1.47 ± 0.78	<0.0001*^Z^

The study found differences in VAS scores both on day one and day seven from the date of surgery (p<0.0001). The pain assessment immediately after the operation revealed a score of 6.55 ±1.19 for the open group and 5.30 ±1.10 for the LigaSure, indicating a lower score for the latter. Similarly, when postoperative pain was assessed after seven days, the LigaSure hemorrhoidectomy group had a lower VAS score of 1.47 ±0.78 compared to a score of 2.25 ±1.26 for the open group (Table 2).

## Discussion

When compared to the traditional Milligan-Morgan method, we found that the LigaSure approach for hemorrhoidectomy results in a shorter duration of operation and a shorter hospital stay for patients. Both immediately post-operation and after a week, the patients reported lesser pain and were found to resume their normal routines earlier. For prolapsed hemorrhoids, hemorrhoidectomy remains the preferred choice of treatment and many surgical techniques have been employed for the same. However, the selection of the most appropriate method to achieve the best operative outcome with minimal complications remains elusive. In this context, our study compared the open and LigaSure hemorrhoidectomy approach and we believe our findings will benefit both surgeons and patients.

Our finding supports the study result of Fareed et al. [18], who recorded a shorter operative time for LigaSure in comparison to closed Ferguson hemorrhoidectomy. Similarly, a prospective randomized clinical trial [19] also reported shorter operative time for LigaSure vs. Milligan-Morgan hemorrhoidectomy. Likewise, the LigaSure hemorrhoidectomy exhibited a shorter operative time as compared to conventional diathermy in a prospective study by Bessa [20]. Thus, the present study reinforces the advantages of using LigaSure hemorrhoidectomy as a speedy alternative amidst many popular surgical techniques for hemorrhoidectomy. The reduction in operative time can be attributed to time saved in achieving hemostasis or ligating the pedicles [20].

In terms of postoperative complications, the LigaSure group had just two, while open hemorrhoidectomy had five problematic instances. Several studies [20-22] comparing LigaSure to other surgical methods found a comparable statistically negligible difference in postoperative problems. Wlodarczyk et al. [23] reported identical risks for bleeding and postoperative sequelae when comparing LigaSure to closed hemorrhoidectomy. The complication of urine retention was not observed in any case for the LigaSure group in our study; however, a prevalence range of 0-11.1% for urine retention has been reported after LigaSure hemorrhoidectomy for chronic hemorrhoids in several studies [1,24,25]. The duration of hospital stay for patients of the LigaSure group was significantly shorter than for open hemorrhoidectomy. This shorter duration in cases of LigaSure hemorrhoidectomy in the present study is a significant improvement as compared to previous studies by Chung and Wu [26] and Sakr [19].

The resumption of work was quicker for the LigaSure group, which aligned with the study by Sakr [19] wherein the open hemorrhoidectomy group took twice the time reported for the LigaSure group to return to normal activities. However, in another clinical study [26], no significant difference in return to normal activities was observed between groups undergoing LigaSure and those with Ferguson closed hemorrhoidectomy.

After seven postoperative days, the LigaSure group's mean pain score was considerably lower than that of the open hemorrhoidectomy group. This is consistent with the findings of a prospective study [20], which found a weekly median pain level of 4.4 and 7.1 for groups undergoing LigaSure and diathermy hemorrhoidectomy, respectively. Other studies [26-28] also show a considerable reduction in pain scores with the LigaSure approach when compared to other conventional hemorrhoidectomy approaches. In contrast, Lee et al. [9] found no difference in VAS scores between groups that received LigaSure or stapled hemorrhoidopexy. Another front for discussion when comparing surgical techniques for the management of hemorrhoidal diseases is the cost involved. While LigaSure is surely heavier on the pocket as compared to the conventional Milligan-Morgan approach, the benefits offered by the former outweigh the financial burden it imposes.

Limitations

While the study highlights the potential benefits of LigaSure hemorrhoidectomy, it is important to acknowledge its limitations, including the small sample size, lack of long-term follow-up, and exclusion of cost analysis. Previously published studies on LigaSure hemorrhoidectomy have employed similar [19-21] or even smaller sample sizes [18,26,28]. We recommend randomized controlled trials with larger sample sizes and longer-term follow-ups so that the superiority of the LigaSure technique in terms of safe and improved operative outcomes is firmly established.

## Conclusions

We found the LigaSure technique to be superior in several key aspects. It demonstrated significantly shorter operation durations, reduced hospital stays, lower pain scores both immediately post-operation and after one week, and enabled patients to return to their daily activities more quickly. These findings align with and reinforce results from previous studies evaluating LigaSure hemorrhoidectomy. The reduced operative time can likely be attributed to the efficient vessel sealing capabilities of the LigaSure device, which minimizes time spent achieving hemostasis. Additionally, the lower postoperative pain scores observed with LigaSure may contribute to faster recovery and earlier hospital discharge.

While our study had some limitations, including the smaller sample size and lack of long-term follow-up, the consistent advantages demonstrated by the LigaSure technique are noteworthy. In light of these findings, surgeons may consider adopting the LigaSure approach for hemorrhoidectomy to potentially improve surgical efficiency, reduce patient discomfort, and enhance overall outcomes. However, further randomized controlled trials with larger patient cohorts and extended follow-up periods would be valuable to definitively establish the long-term benefits of LigaSure hemorrhoidectomy in routine surgical practice for the management of advanced hemorrhoidal disease.
